# Acute Cytomegalovirus Infection as a Cause of Venous Thromboembolism

**DOI:** 10.4084/MJHID.2014.041

**Published:** 2014-06-01

**Authors:** Francesca Rinaldi, Raffaella Lissandrin, Francesco Mojoli, Fausto Baldanti, Enrico Brunetti, Michela Pascarella, Maria Teresa Giordani

**Affiliations:** 1 Department of Infectious Diseases, IRCCS San Matteo Hospital Foundation, University of Pavia, Viale Golgi 19, 27100 Pavia, Italy; 2 Clinical-Surgical, Diagnostic and Pediatric Sciences Department, Section of Anesthesia and Intensive Care 1, University of Pavia, IRCCS San Matteo Hospital Foundation, Viale Golgi 19, 27100 Pavia, Italy; 3 Molecular Virology Unit, Virology and Microbiology Department, IRCCS San Matteo Hospital Foundation, Viale Golgi 19, 27100 Pavia, Italy; 4 Microbiology Service, San Bortolo Hospital, Viale Rodolfi 37, 36100 Vicenza, Italy; 5 Infectious and Tropical Diseases Unit, San Bortolo Hospital, Viale Rodolfi 37, 36100 Vicenza, Italy

## Abstract

Acute Human Cytomegalovirus (HCMV) infection is an unusual cause of venous thromboembolism, a potentially life-threatening condition. Thrombus formation can occur at the onset of the disease or later during the recovery and may also occur in the absence of acute HCMV hepatitis. It is likely due to both vascular endothelium damage caused by HCMV and impairment of the clotting balance caused by the virus itself. Here we report on two immunocompetent women with splanchnic thrombosis that occurred during the course of acute HCMV infection. Although the prevalence of venous thrombosis in patients with acute HCMV infection is unknown, physicians should be aware of its occurrence, particularly in immunocompetent patients presenting with fever and unexplained abdominal pain.

## Introduction

Acute portal vein thrombosis (PVT) is clinically relevant condition usually associated with bowel ischemia caused by the involvement of superior mesenteric and splenic veins. In the general population, the prevalence of PVT is about 1% but can reach 16–40% in patients with cirrhosis or cancer.^1^ In more than 70% of cases, PVT is associated with local factors, such as cirrhosis, malignancy, intra-abdominal infection, abdominal surgery, trauma or liver transplantation. Moreover, up to 72% of presumed idiopathic PVT may be associated with thrombophilic conditions and hematologic diseases.^2^ PVT has been described in patients with acute hepatitis caused by EBV, HAV, HBV, HCV, but it has been more often associated with acute HCMV hepatitis.^3^ In this report, we describe two cases of acute PVT associated with an HCMV acute infection in two adult immunocompetent women.

## Case One

A 62-year-old female was admitted to San Bortolo Hospital, Vicenza, Italy, in September 2011, with fever and abdominal pain lasting for several weeks. The patient’s and family history were unremarkable. Physical examination revealed only slight discomfort on palpation of the epigastric area. Liver and spleen were regular in size.

White blood count (WBC) was 11.7 × 10^3^/μL, 60% lymphocytes, and liver function tests (LFT) were abnormal (AST 333 IU/mL, ALT 433 IU/mL [normal value <37], GGT 296 IU/mL [n.v. <33], alkaline phosphatase (ALP) 221 IU/L [normal range 30–105], bilirubin 1.0 mg/dL [n.v. <1.3]), some inflammatory indices were raised (C-reactive Protein 3.65 mg/L [n.r 0.00–0.50], ferritin 1442 ng/mL [n.r. 10–160 ], lactate dehydrogenase 1098 U/L [n.r. 200–420], creatine phosphokinase 24 U/L [n.r. <200 ] procalcitonin 0.54 ng/mL [n.v.<0.5], with normal erythrocyte sedimentation rate (ESR) 27 mm/h [n.r. 2–45]).

**Figure 1:**
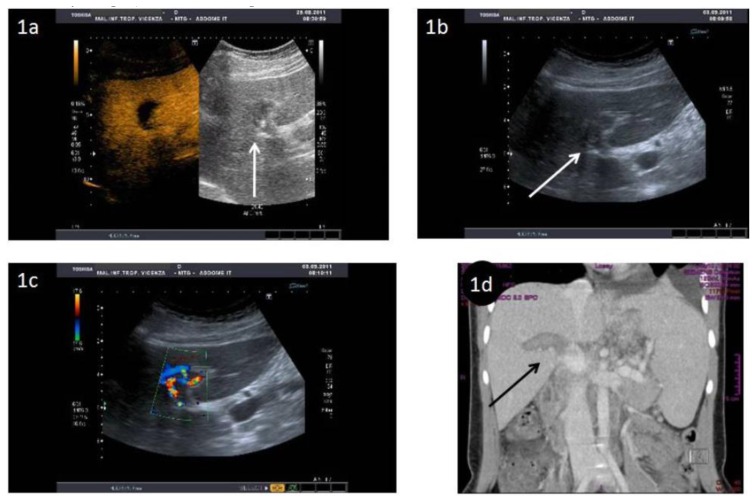
**1a-** Patient One: CEUS and US on admission, showing the thrombus in the left portal branch (arrow). **1b-** Patient One: US on discharge, showing the thrombus organization (retraction and hyperechoic clot) (arrow). **1c-** Patient One: US-Doppler on discharge, showing partial revascularization. **1d-** Patient Two: coronal scan of abdominal contrast media CT. The hypodense material is inside the portal vein (arrow) and the spleen is enlarged.

Chest X-Ray was negative. An abdominal ultrasound (US) (Aplio XG Model SSA-790A, Toshiba, Tokyo, Japan), showed a complete thrombosis of the left portal branch, confirmed by a contrast-enhanced ultrasound (SonoVue, Bracco, the Netherlands) and a computed tomography (CT) scan (see Figure 1, Panel 1a). No other lesions were found in the abdomen. A hematologic evaluation ruled out myeloproliferative and lymphoproliferative disorders; although a bone-marrow biopsy was not considered, an immune-phenotypic analysis of the peripheral white blood cells and red blood cells resulted in normal range and did not find cellular clones with GPI-linked molecules deficit (in order to exclude paroxysmal nocturnal hemoglobinuria). Inherited and acquired risk factors for thrombosis were ruled out (See Table 1). The patient was a mild smoker (5 cigarettes/day) and was not on hormones; neoplastic markers (Carcinoembryonic [CEA] and carbohydrate antigen [Ca] 19.9 and 15.3, alpha-fetoprotein [AFP]) were all negative. Antibodies against the main hepatotropic viruses (HAV, HBV, HCV, HEV) were negative while HCMV serology suggested primary infection (IgM 10.86 mg/dL [negative <1], IgG 12.8 mg/dL [negative<11]). HCMV DNA (real-time PCR, Real Time Alert QPCR, Elitech Group Nanogen, Milan, Italy) was positive at 3434 copies/ml (sensitivity cutoff: >1111). Thus, acute HCMV hepatitis complicated with portal vein thrombosis was hypothesized.

The patient was treated with i.v. gancyclovir (5 mg/Kg/bid) and low molecular weight heparin (sodium enoxaparin 4.000 U.I. bid) for 15 days. On discharge, HCMV DNA was undetectable. An abdominal US performed before discharge showed thrombus organization (retraction and hyperechoic clot) (see Figure 1, Panels 1b and 1c). The patient continued oral anticoagulant treatment (Warfarin 5 mg/day with INR check every 2–3 days in the first two weeks) for 21 months after discharge until her abdominal US showed an almost complete re-canalization of the left portal branch. A prolonged follow-up (40 months) including clinical interview, abdominal ultrasound, blood and LFT resulted normal.

## Case Two

A 20-year-old Caucasian woman was admitted to Maggiore Hospital in Crema, Italy, in November 2011, for gastric pain and headache lasting three days. A US scan of the upper abdomen (Esaote myLab25, Esaote, Genova, Italy) showed portal thrombosis of the intra-hepatic right and left branches, splenomegaly and free fluid in the perihepatic area and in the Douglas pouch. Chest X-Ray was negative. Biochemical examination revealed abnormal liver function tests: (AST 162 IU/ml, ALT 243 IU/mL, GGT 167 IU/mL, ALP 402 IU/L) and increased inflammatory indices (Lactate dehydrogenase 747 U/L, C-reactive Protein 1.5 mg/L); ESR was normal at 23 mm/h.

The patient received anticoagulant therapy (continuous infusion of unfractionated heparin, 18 UI/Kg/h). An abdominal CT confirmed portal thrombosis involving the left and the right intra-hepatic branches and the superior and inferior mesenteric veins. A 3 cm long thrombus was identified at the beginning of the splenic vein, which was still patent. The patient was transferred to the Intensive Care (ICU) of San Matteo Hospital, Pavia, Unit where blood tests confirmed abnormal liver enzymes with normal white blood cells (WBC: 4100/mm^3^, 63% of lymphocytes). At ICU admission, a new CT scan confirmed the thrombosis (see Figure 1, Panel 1d) and showed also pericardial and bilateral pleural effusions.

Thrombophilic conditions were ruled out by specific tests (See Table 1). The patient did not smoke and was not on hormones.

Lymphocyte immunophenotyping was consistent with acute viral illness (CD4/CD8 0.5, CD4: 31.1%, CD8: 57.8%). Serology revealed acute HCMV infection: IgM anti CMV (ELISA): 11.8 (negative < 0.9, positive > 1.1), IgG avidity test (ELISA): 18%, (low < 35, high >50). HCMV DNA (real-time PCR) was positive 100 copies/ml (cut-off ≥100 copies). Serology for HIV, HCV, HBV, *S.typhi*, *Yersinia* spp, Hantavirus, *Listeria* spp, *Leptospira* spp, *Borrelia* spp and HSV-8 were negative. Neoplastic markers (CEA, Ca 19.9 and Ca15.3, AFP) were all negative.

She was treated with anticoagulants (unfractionated heparin followed by enoxaparin 100 UI/Kg), with progressive regression of the thrombosis and disappearance of the fluid collections. A US scan one month after discharge showed further regression of PVT.

## Discussion

Although HCMV infection is very common, up to 40% of adult immunocompetent subjects are seronegative for HCMV. The prevalence of HCMV infection is underestimated, since it occurs in childhood when it is usually asymptomatic. In the adult it is frequently characterized by fever, swollen neck lymph nodes and mild hepatitis, spleen enlargement and low platelet count. The true rate of symptomatic disease is, however, unknown. The diagnosis is commonly made by serology (IgM anti-CMV positivity and rising title of IgG anti-CMV).

HCMV reactivation is commonly seen in immunocompromised individuals such as transplant and AIDS patients, leukemia and lymphomas, or in situations such as prolonged steroids administration for rheumatologic and autoimmune diseases. Portal thrombosis is an uncommon but not so rare event occurring during acute HCMV infection and has been recently sporadically documented.^4^,^5^ Atzmony and Abgueguen in 2010 reported a prevalence of thrombosis among hospitalized patients with acute HCMV infection of about 6.4% and 7.9% respectively, while Justo et al. reported a 1.9–9.1% incidence of HCMV infection in patients with thrombotic events.^6^,^7^,^4^ HCMV-related thrombosis can occur also several weeks after the diagnosis of acute HCMV.^7^

Unlike other HCMV lesions, which differ in immunocompetent and immunocompromised patients, deep venous thrombosis seems to occur in both categories of patients,^8^ but with different localization. Justo et al. in 2011 showed how immunocompetent patients experienced more splanchnic vein thrombosis while HIV immunocompromised ones are more susceptible to deep vein thrombosis and pulmonary embolism during highly active antiretroviral treatment (HAART).^4^

Several mechanisms have been proposed to explain HCMV-induced vascular thrombosis, including a direct action by HCMV itself and the activation of different molecules. HCMV could directly invade the endothelial cells, causing endothelial damage and the activation of coagulation factors.^9^ It could also facilitate thrombin production and interrupt the synthesis of prostaglandin and interleukin-2 due to procoagulant phospholipids on its surface. Both these mechanisms cause platelet adhesion.^9^

HCMV, like other viruses and bacteria, seem to induce the production of anti-phospholipid antibodies 10 and can inhibit P53-mediated apoptosis through the activation of its first gene transcribed, IE84, which binds P53 and inhibits its transcriptional activity.^7^

Predisposition to thrombosis can be linked to different clinical situations, such as the use of oral contraceptives, surgery, pregnancy, active malignancy (directly or as part of paraneoplastic syndrome), and protracted immobility.^4^,^11^ All these factors seem to be prevalent in immunocompetent patients.^4^ In hematological disorders, causes of vein thrombosis are transient or permanent antiphospholipid syndrome, factor V Leiden heterozygote mutation, protein C or protein S deficiency. In a recent literature review, chronic myeloproliferative neoplasms (MPN) are associated in 6–33% of patients with portal vein thrombosis.^2^ In the review, the JAK2 V617F somatic mutation was reported both in overt and non-overt MPN.^2^ Coagulation factors defects and myeloproliferative disorders, including JAK2 V617F somatic mutation had been ruled out in both cases (see Table 1).

The clinical manifestations of acute portal vein thrombosis (PVT) depend on the extent of the obstruction and the speed of its development. The consequences of an untreated PVT depend on the same factors: if portal flow is suddenly interrupted (as happens in experimental thrombosis) a liver infarction or an ischemia of the bowel may follow.

Even incomplete thrombosis can alter intestinal permeability and lead to bacterial sepsis, enteric bleeding or diarrhea. In the absence of treatment, thrombus organization can lead to chronic thrombosis and portal cavernoma.

When PVT occurs, anticoagulation therapy for at least 3 months is recommended, starting with low molecular weight heparin in order to achieve rapid anticoagulation and shifting to oral anticoagulation as soon as the patient’s condition has stabilized. Long-term anticoagulation therapy in patients with acute PVT and permanent thrombotic risk factors that are not otherwise correctable is recommended.^11^

Antiviral treatment is mandatory in immunocompromised patients, while its administration in immunocompetent patients is debatable. In case one, HCMV-DNA tested positive, and the clinician decided to treat the patient with gancyclovir. Moreover, the initial suspicion of an associate neoplasm or an hematological malignance played a role in this decision. In case two the viremia was borderline due to the immune system control, so in spite of the more aggressive presentation, virus infection was left untreated.

Finally, bedside US (or “point of care US”) may prove to be a life-saving approach in emergency settings; a protocol finalized to achieve a prompt diagnosis of the thrombotic events can be easily learned by physicians with no previous experience in US.^12^ In our experience, bedside abdominal US can help recognize portal vein thrombosis due to infectious agents like HCMV and avoid its dangerous consequences.

## Conclusions

Physicians should consider hematologic, neoplastic and infectious etiologies in the workup of patients with fever and PVT. Although venous thrombosis during acute HCMV infection is uncommon in immunocompetent patients, physicians should maintain a high index of suspicion, and testing for HCMV IgM in these cases is recommended.

## Figures and Tables

**Table 1:** Laboratory tests

Coagulation Parameters:	Patient 1	Patient 2	Normal values
Prothrombin time (PT)	0.94	0.93	0.7–1
Activated partial thromboplastin time (PTT), sec			20–32
Prothrombin time international normalized ratio (INR)	1	1.02	<1.2
Fibrinogen (mg/dL)	242	286	200–400
Serum cross-linked fibrin (XDP)	233	N.A.	<0.20
Inherited prothrombotic disorders:			
Protein C, %	100	104	70–140
Protein S, %	56	68	53–100
AT Mutation, %	87	75	70–120
Factor V Leiden Mutation (R506Q)	Absent	Absent	Absent
Factor II Mutation (G20210A)	Absent	Absent	Absent
MTHFR (Homocysteine) Mutation	Absent	Absent	Absent
Acquired prothrombotic disorders:			
Jak 2 (Val617Phe)	Absent	Absent	Absent
Anticardiolipin Ab	Absent	Absent	Absent
LA1 (DRV) (sec)	0.8	39.5	<45

N.A. Not available.
